# Tandospirone, a 5-HT_1A_ partial agonist, ameliorates aberrant lactate production in the prefrontal cortex of rats exposed to blockade of N-methy-D-aspartate receptors; Toward the therapeutics of cognitive impairment of schizophrenia

**DOI:** 10.3389/fnbeh.2014.00291

**Published:** 2014-09-03

**Authors:** Takashi Uehara, Tadasu Matsuoka, Tomiki Sumiyoshi

**Affiliations:** ^1^Department of Neuropsychiatry, Kanazawa Medical University, Ucninada-choIshikawa, Japan; ^2^Department of Psychiatry, Matsuoka HospitalOyabe, Toyama, Japan; ^3^Department of Clinical Research Promotion, National Center Hospital, National Center of Neurology and PsychiatryTokyo, Japan

**Keywords:** 5-HT_1A_, cognition, schizophrenia, lactate, microdialysis, glutamate, NMDA receptor, animal model

## Abstract

**Rationale:** Augmentation therapy with serotonin-1A (5-HT_1A_) receptor partial agonists has been suggested to improve cognitive impairment in patients with schizophrenia. Decreased activity of prefrontal cortex may provide a basis for cognitive deficits of the disease. Lactate plays a significant role in the supply of energy to the brain, and glutamatergic neurotransmission contributes to lactate production.

**Objectives and methods:** The purposes of this study were to examine the effect of repeated administration (once a daily for 4 days) of tandospirone (0.05 or 5 mg/kg) on brain energy metabolism, as represented by extracellular lactate concentration (eLAC) in the medial prefrontal cortex (mPFC) of a rat model of schizophrenia.

**Results:** Four-day treatment with MK-801, an NMDA-R antagonist, prolonged eLAC elevation induced by foot-shock stress (FS). Co-administration with the high-dose tandospirone suppressed prolonged FS-induced eLAC elevation in rats receiving MK-801, whereas tandospirone by itself did not affected eLAC increment.

**Conclusions:** These results suggest that stimulation of 5-HT_1A_ receptors ameliorates abnormalities of energy metabolism in the mPFC due to blockade of NMDA receptors. These findings provide a possible mechanism, based on brain energy metabolism, by which 5-HT_1A_ agonism improve cognitive impairment of schizophrenia and related disorders.

## Introduction

Disturbances of cognitive function, evaluated by psychological and neurophysiological methods, have been shown to predict social outcome in patients with schizophrenia (Meltzer and McGurk, 1999; McGurk and Meltzer, 2000). There is much attention to the role of psychotropic compounds acting on serotonin (5-HT) receptors in ameliorating cognitive deficits of the disease. Among the 5-HT receptor subtypes, the 5-HT_1A_ receptor is attracting particular interests as a potential target for enhancing cognition (Newman-Tancredi and Albert, 2012; Ohno et al., 2012; Sumiyoshi and Higuchi, 2013; Sumiyoshi et al., 2013). It is reported that adjunctive treatment with selective 5-HT_1A_ receptor (partial) agonists, e.g., tandospirone or buspirone, was associated with improvements in some types of cognitive function in patients with schizophrenia (Sumiyoshi et al., 2001b, 2007; Sumiyoshi and Higuchi, 2013). These observations provide the basis for the ability of 5-HT_1A_ receptor stimulation to enhance cognition, a therapeutic approach that have promoted the development of novel antipsychotic drugs (Sumiyoshi, 2013; Sumiyoshi et al., 2013).

The development of animal models of schizophrenia is important to clarify the pathophysiology of the illness and facilitate the development of novel therapeutics. Non-competitive antagonists at the N-methyl-D-asparate receptor (NMDA-R) have been shown to induce schizophrenia-like symptoms, i.e., positive and negative symptoms, as well as cognitive dysfunction in normal subjects (Jentsch and Roth, 1999). Numerous studies reported that NMDA-R antagonists, such as phencyclidine (PCP), MK-801 and ketamine, produce hyperlocomotion, stereotypy, information processing deficits, impairments of cognitive functions and social interactions, behavioral changes reminiscent of symptoms of schizophrenia (Breese et al., 2002; Moghaddam and Jackson, 2003; Bubenikova-Valesova et al., 2008; Jones et al., 2011).

Four-day treatment with MK-801 changed behaviors and expression of NMDA-Rs in a way that mimicked chronic treatment (Bubenikova-Valesova et al., 2010). Especially, prepulse inhibition (PPI) was impaired, while locomotion was decreased (Bubenikova-Valesova et al., 2010). PPI deficits are one of the most widely used neurophysiological markers of the pathophysiology of schizophrenia, and have been suggested to represent an aspect of cognitive deficits (Geyer, 2006; Singer et al., 2013). Especially, a specific correlative link between working memory and PPI has been reported in rodents (Singer et al., 2013). On the other hand, the mPFC was the target brain region in the current study because our aim was to clarify the mechanisms underlying cognitive enhancement by 5-HT_1A_ receptor stimulation. Functional abnormality of the frontal cortex has been associated with negative symptoms and cognitive deficits (Volk and Lewis, 2002). Specifically, performance on the cognitive tasks governed by the prefrontal cortex (PFC), e.g., working memory and executive function, has been consistently reported in patients with schizophrenia (Tamminga et al., 1992; Volz et al., 1999; Hazlett et al., 2000; Ragland et al., 2007).

Since the proposal of the astrocyte-neuron lactate shuttle (ANLS) hypothesis (Pellerin and Magistretti, 1994), lactate has been found to play a crucial role in energy metabolism in the brain (Tsacopoulos and Magistretti, 1996; Pellerin and Magistretti, 2012). According to this hypothesis, lactate is produced in a neural activity-dependent and glutamate-mediated manner in astrocytes, and is transferred to active neurons (Pellerin et al., 1998, 2007; Pellerin, 2003; Uehara et al., 2008). Moreover, it has been shown that lactate *is* a primary substrate for energy metabolism in the brain of humans (Smith et al., 2003) or rodents (Wyss et al., 2011) under working conditions if both lactate and glucose is sufficiently available, as demonstrated by *in vivo* studies (Wyss et al., 2011).

Whether or not abnormality of lactate metabolism exists in the brain of schizophrenia patients remains to be discussed. A postmortem study reports altered transcription of genes in a large number of metabolic pathways and increased lactate levels in the PFC of patients with schizophrenia (Prabakaran et al., 2004). However, another report argued that these changes are associated with decreased pH, and are possibly related to antipsychotic treatment rather than a primary metabolic abnormality (Halim et al., 2008).

In this study, we investigated the effect of repeated (four consecutive days) administration of MK-801, a non-competitive NMDA-R antagonist, on energy metabolism indicated by extracellular lactate concentration (eLAC) in the medial prefrontal cortex (mPFC) of rats using microdialysis technique. Then, we determined whether co-administration of low and high doses (0.05 or 5 mg/kg) tandospirone affect lactate metabolism in the MK-801 treated animals.

## Materials and methods

### Animals

Adult male Wistar rats (Japan SLC Inc., Hamamatsu, Japan) weighing 280–300 g (8 weeks postnatal) for microdialysis experiments were housed 4–5 in a standard cage at 24 ± 2°C under a 12-h light (07:00–19:00 h) 12-h dark cycle. The procedures complied with the National Institutes of Health guide for the care and use of Laboratory animals. All experiments were reviewed and approved by the Committee of Animal Research, University of Toyama.

### Drugs

Tandospirone was provided from Dainippon Sumitomo Pharmaceuticals (Tokyo, Japan).

**Figure 1:**
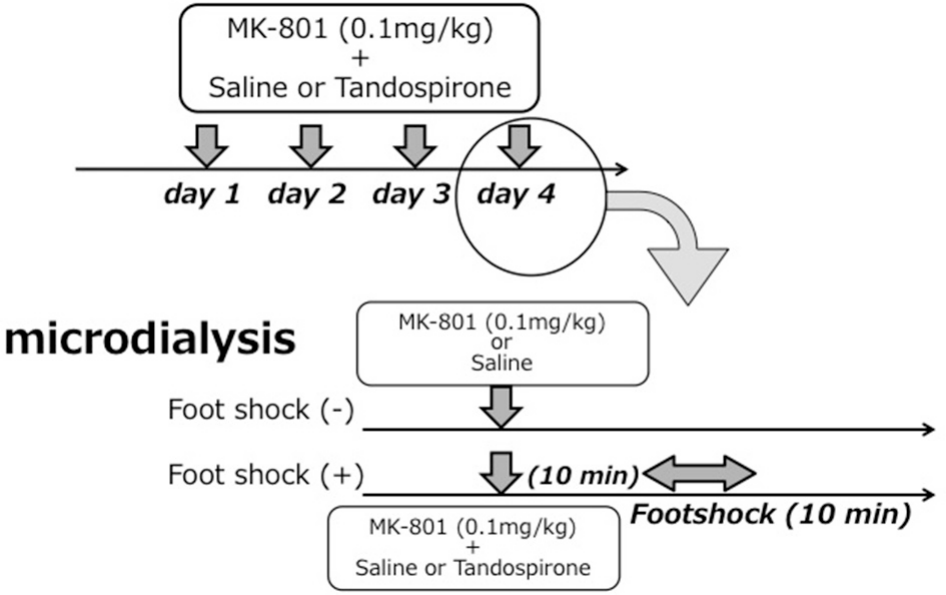
**Schematic representation of the experimental protocol for microdialysis experiments**. Upper parts in each figure reveals drug treatment for 4 days and lower parts time course in the 4th day (experiment day).

The treatment regimen with MK-801 (dizocilpine, 0.1 mg/kg, Sigma-Aldrich, St. Louis, MO) and/or tandospirone was based on a previous report (Bubenikova-Valesova et al., 2010) with minor modifications. First, to evaluate the effect of 4 days treatment with MK-801 on eLAC in the mPFC, rats received an intraperitoneal (i.p.) injection of MK-801 (0.1 mg/kg) at a volume of 2 ml/kg (MK-801 group, *n* = 6), or the equal volume of saline (saline group, *n* = 5) once daily for 4 days. Second, to access the effect of tandospirone on FS-induced eLAC changes, rats received a subcutaneous (s.c.) injection of tandospirone at 0.05 mg/kg (low-T) or 5.0 mg/kg (high-T) at a volume of 2 ml/kg or the equal volume of saline. This yielded the following 6 groups: saline-saline group (*n* = 5); saline-low-T group (*n* = 5); saline-high-T group (*n* = 5); MK-801-saline group (*n* = 5); MK-801-low-T group (n–5); MK-801-high-T group (*n* = 5). The last injection was administered 10 min before the start of foot-shock (FS) stress (Figure 1).

### Surgery

Extracellular lactate concentrations were measured by *in vivo* microdialysis technique with an enzyme reactor/fluorometric detector according to the method previously reported (Uehara et al., 2003, 2005, 2006, 2007a). The animals were anesthetized with pentobarbital sodium (Nembutal, Abbott Laboratories, IL, USA) (40 mg/kg, i.p.), and were mounted on a stereotaxic apparatus. A dialysis probe (molecular weight cutoff 10,000; 200 μm in outer diameter) was implanted into the left mPFC according to the atlas of Paxinos and Watson (1998), and was secured with skull screws and dental acrylate. The exposed tip length of the probe was 1.5 mm. Coordinates were A 3.2 mm, L 0.6 mm, V 5.2 mm from bregma for the mPFC. Following the surgery, the rats were housed in individual cages with free access to food and water.

Forty-two to 48 h after surgery, the dialysis experiment was carried out on the freely moving rats. Microdialysis experiments were performed between 08:00 and 16:00. Artificial CSF (consisting of 147 mmol/l NaCl, 3 mmol/l KCl, 1.2 mmol/l CaCl_2_, 1.2 mmol/l MgSO_4_, 0.4 mmol/l NaH_2_PO_2_, pH 7.40) was perfused at a rate of 5.0 μl/min into the dialysis probe. The dialysates were mixed on-line with an enzyme solution containing L-lactate dehydrogenase and NAD^+^ in a T-tube. The enzyme solution consisted of 5.0 μg/ml LDH (L-Malate: NAD oxidoreductase, E.C.1.1.1.27; isolated from pig heart, specific activity ca.300 U/mg; Roche Diagnostics GmbH, Mannheim, Germany) and 0.5 mmol/l NAD^+^ (Roche Diagnostics GmbH, Mannheim, Germany) in a carbonate buffer (62.5 mmol/l, adjusted to pH 9.4 with NaOH). The solution was pumped using a Model 22 microdialysis pump (Harvard Apparatus, Inc. Massachusetts, USA) at the flow rate of 20 μl/min. The mixture from the T-tube was passed for 20 min reaction before reaching a fluorometer equipped with a 12 μl flow-cell (Shimazu RF-530, Kyoto, Japan). During transport to the fluorometer, lactate was enzymatically oxidized, and the fluorescence of the formed nicotinamid adenosine dinucleotide diphosphate (NADH) was continuously measured with excitation at 340 nm and emission at 450 nm. A standard solution of 100 μmol/l lactate was used for calibration.

### Foot-shock stress

Foot-shock (FS) stress was administered using a plastic communication box (CB5, MATYS, Toyama, Japan), according to the method reported previously (Uehara et al., 2012a). The box (L 51 × W 51 × H 40 cm) was equipped with a grid floor composed of stainless steel rods (6-mm in diameter) placed 16 mm apart. The box was subdivided into nine compartments (17 × 17 cm) by transparent plastic walls. In this study, we used 4 compartments areas (34 × 34 cm) for the field of free moving and FS administration. The communication box was connected to a shock-generator (MSG-001, MATYS, Toyama, Japan) and a timer-box (MTB-001, MATYS, Toyama, Japan) to deliver FS as described below. Each FS session consisted of a scramble shock of 0.3 mA for 5 s administered every 30 s for 10 min.

At the end of the experimental sessions, all rats were deeply anesthetized with pentobarbital sodium, and were sacrificed by decapitation. The position of dialysis probes was verified microscopically for all rats.

### Presentation of the results and statistics

Data were analyzed by analysis of variance (ANOVA) using SPSS software (version 19.0 J for Mac; IBM, Tokyo, Japan).

Lactate levels in the dialysates are expressed as μmol/l calculated by a standard solution of 100 μmol/l lactate. The average of the eLAC during the period preceding the last injection (four measurements performed every 5 min) was used as the basal concentrations of lactate. To access the effects of MK-801 and tandospirone on basal lactate levels, two-way ANOVA were carried out with MK-801 treatment (saline, MK-801) as one factor and tandospirone treatment (saline, low-T, high-T) as the second factor. Data from MK-801 administration was analyzed using repeated measure ANOVA. MK-801 treatment (saline, MK-801). Time was treated as repeated measures variable. Data from FS stress procedures were analyzed using two-way repeated measure ANOVA. MK-801 treatment (saline, MK-801) and tandospirone treatment (saline, low-T, high-T) were treated as between-group variable. Time was treated as repeated measures variable. Thereafter, two-way repeated measures ANOVA were performed for each group. Data were analyzed by Kruskal-Wallis test at each time point, and statistical significance was further examined by Mann-Whitney test for multiple comparisons.

To compare the results of with and without FS experiments, we analyzed total increase amount of lactate after last injection in each groups calculated by sum of eLAC data subtractiong the basal concentration of eLAC from eLAC concentration at each time point (from time 0 to time 110). These data were analyzed using one-factor ANOVA (8 groups; saline group without FS, MK-801 group without FS, saline-saline group, saline-low-T group, saline-high-T group, MK-801-saline group, MK-801-low-T group, MK-801-high-T group) followed by *post-hoc* Bonferroni test. A probability (P) of less than 0.05 was considered to be significant.

## Results

### Basal lactate levels

The basal concentrations of lactate in 8 groups (with or without FS experiments) were shown in Table 1. There were no significant interaction [*F*_(2,35)_ = 0.48, *P* = 0.62], main effect of MK-801 treatment [*F*_(1,35)_ = 0.48, *P* = 0.49] and tandospirone treatment [*F*_(2,35)_ = 0.19, *P* = 0.83].

**Table 1:** **The basal concentrations of lactate in dailysates in each grop**.

	**Foot-shock stress(−)**	**Foot-shock stress (+)**		
		**Saline**	**Tandospirone**	
			**Low (0.05 mg/kg)**	**High (5.0 mg/kg)**
Saline	50.92 ± 4.99	47.26 ± 4.99	51.46 ± 2.44	50.44 ± 2.63
MK-801	50.47 ± 1.20	47.62 ± 5.12	48.23 ± 2.5	43.96 ± 7.26

*The basal concentrations of lactate in daily sates were presented the average of the eLAC during the period preceding the last injection (four measurements performed every 5 min). Values are expressed as mean ± SE, μmol/L*.

### Effect of MK-801 administration on eLAC

**Figure 2:**
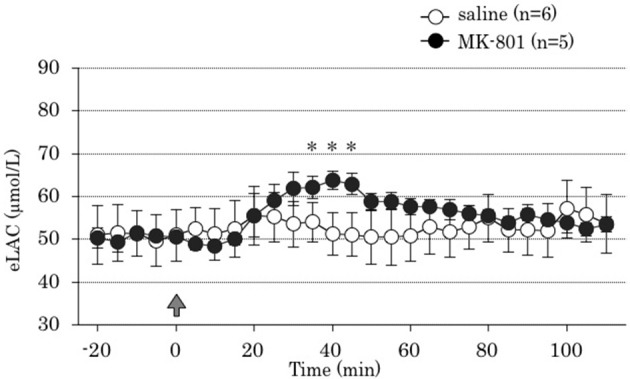
**Time course of the effect of 4 days treatment of MK-801 on the extracellular lactate concentrations (eLAC) in the medial prefrontal cortex**. Rats were treated with saline (saline group, *n* = 6; open circle) or MK-801 (MK-801 group, *n* = 5; closed circle). Lactate levels in the dialysates are expressed as μmol/l calculated by a standard solution of 100 μmol/l lactate. Data are mean ± s.e.m. The arrow indicates the timing of the last injection. Asterisk showed *p* < 0.05 vs. saline group in each time point.

Repeated measures ANOVA demonstrated that MK-801 treatment increased eLAC in the mPFC [MK-801 x time interaction, *F*_(22,198)_ = 5.73, *P* < 0.001]. MK-801 administration increased eLAC from 35 to 45 min (Figure 2).

### Effect of MK-801 and tandospirone combination on FS-induced eLAC elevation

**Figure 3:**
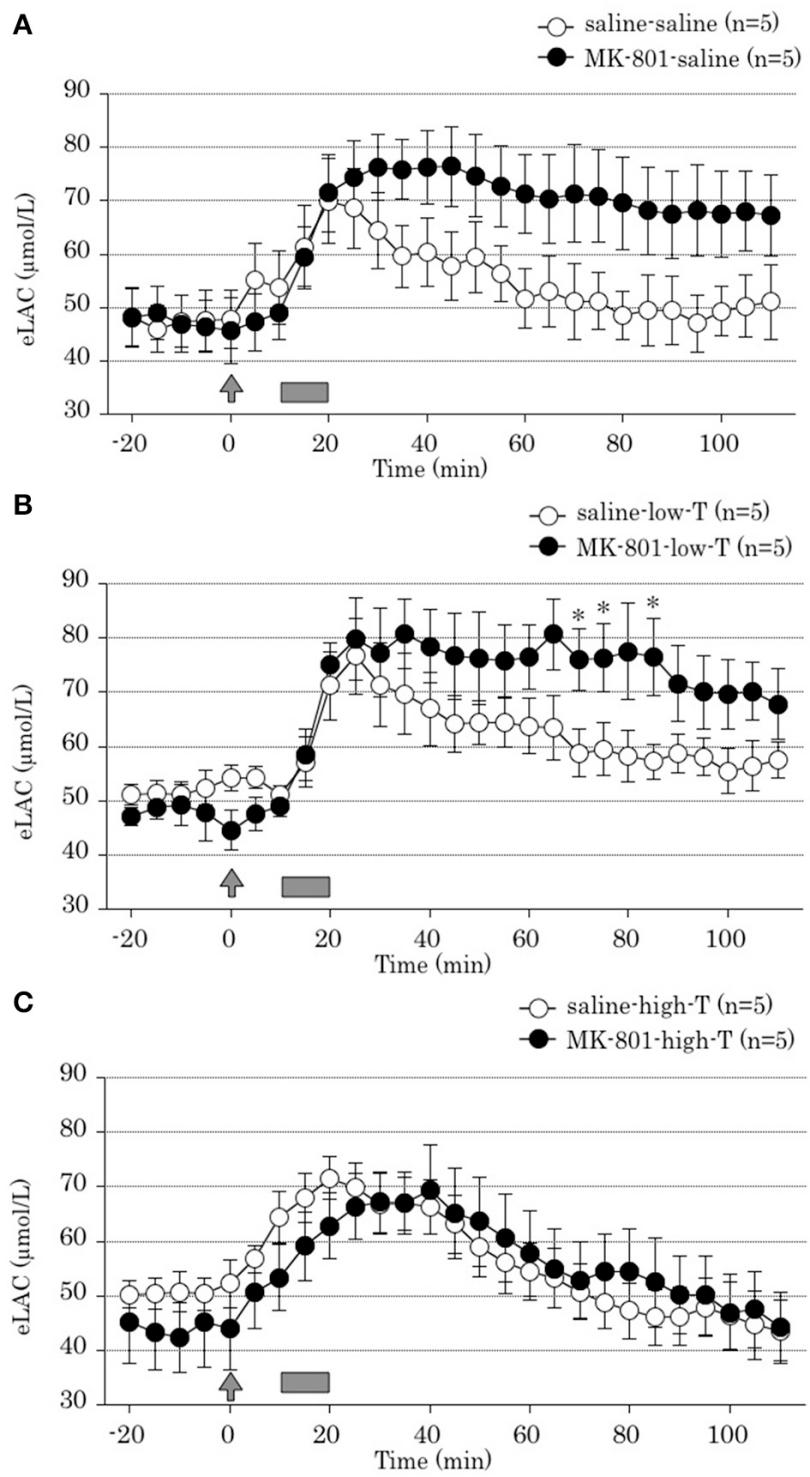
**Effect of tandospirone on foot-shock stress-induced increment of the extracellular lactate concentrations (eLAC) in the medial prefrontal cortex in rats treated with (A) saline (saline-saline group, *n* = 5; MK-801-saline group, *n* = 5), (B) tandospirone 0.05 mg/kg (saline-low-T group, *n* = 5; MK-801-low-T group, n-5) and (C) 5.0 mg/kg tandospirone (saline-high-T group, *n* = 5; MK-801-high-T group, *n* = 5)**. Rats were simultaneously treated with saline (open circle) or MK-801 (closed circle), respectively. Lactate levels in the dialysates are expressed as μmol/l calculated by a standard solution of 100 μmol/l lactate. Data are mean ± s.e.m. The arrow indicates the timing of the last injection. Foot-shock is indicated by solid bars. Asterisk showed *p* < 0.05 vs. saline group in each time point.

Two-way ANOVA revealed that both MK-801 treatment x time interaction and tandospirone treatment x time interaction were significant [*F*_(22,528)_ = 10.31, *P* < 0.001; *F*_(44,528)_ = 5.65, *P* < 0.001; respectively]. These results indicated that MK-801 treatment produced prolonged FS-induced eLAC elevation (Figure 3A), and that co-administration with tandospirone (5.0 mg/kg) abolished this change (Figure 3C) but not tandospirone (0.05 mg/kg) (Figure 3B). At each time point data analysis, there were significant eLAC differences between MK-801 and saline group at 70, 75, and 85 min after the last injection in low-dose tandospirone administration group (Figure 3B), whereas no significant differences at each time point in saline or high-dose tandospirone administration group (Figures 3A,C).

### Effect of tandospirone on FS-induced eLAC elevation in saline treatment group

Two-way ANOVA revealed no significant tandospirone treatment x time interaction [*F*_(44,264)_ = 1.90, *P* = 0.08]. This result demonstrated that 4 days treatment with tandospirone did not affected on FS-induced eLAC elevation in the mPFC (Figures 3A,C).

### Total increase amount of lactate after last injection

**Figure 4:**
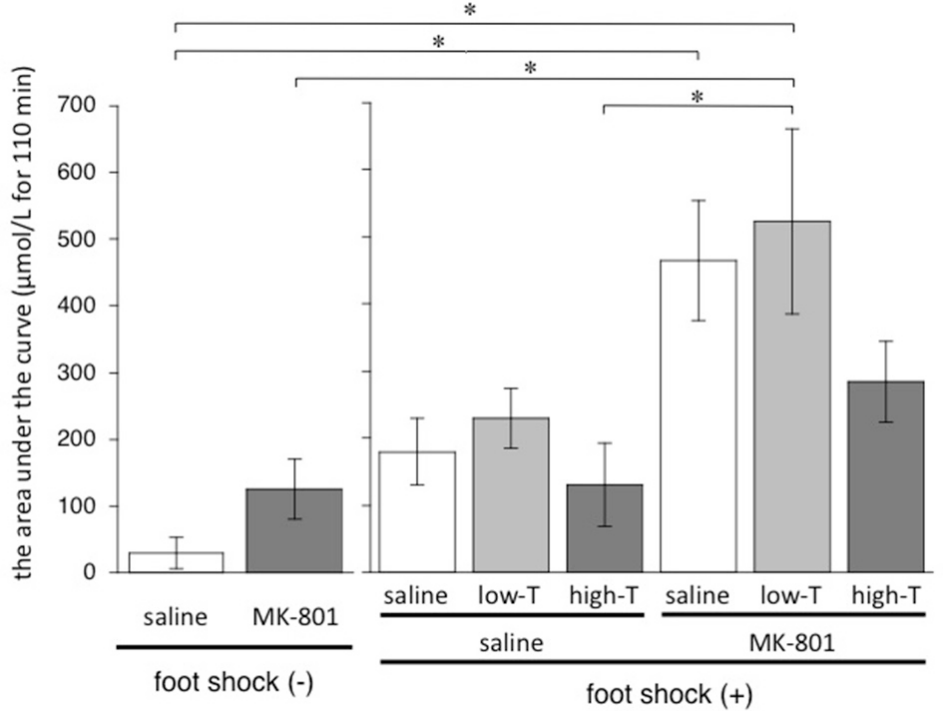
**Total increase of lactate after injection (from time 0 to time 110 min) calculated by sum of (each eLAC concentration—basal concentration of eLAC)**. Basal concentration of eLAC is the average of eLAC during the period preceding the last injection (four measurements performed every 5 min). Asterisk showed *p* < 0.05 between groups.

One-factor ANOVA demonstrated significant differences among 8 groups [*F*_(7,33)_ = 6.01, *P* < 0.001]. *Post-hoc* Bonferroni test showed significant differences between saline group without FS and MK-801-saline group, saline group without FS and MK-801-low-T group, MK-801 group without FS and MK-801-low-T group, saline-high-T group and MK-801-low-T group (Figure 4). These results indicated that repeated MK-801 administration by itself have a temporary effect on eLAC changes in the mPFC.

## Discussion

This study obtained results showing; (1) acute treatment with MK-801 in the 4th day increased eLAC to around 65 μmol/L above the base line level, and 4-day treatment with MK-801 prolonged eLAC elevation induced by FS in the mPFC, whereas its effect was temporary, and (2) co-administration with high-dose tandospirone abolished the effect of MK-801 treatment on prolonged eLAC elevation.

Four-day treatment with MK-801 at the young adult stage resulted in increasing of eLAC after FS (Figure 3), while repeated MK-801 treatment by itself may have little effect on total increase amount of lactate (Figure 4). This finding is consistent with a postmortem study reporting an increase in lactate levels in the PFC of patients with schizophrenia (Prabakaran et al., 2004). Possible mechanisms include glutamatergic neurotransmissions, because eLAC has been suggested to be linked to them (Uehara et al., 2008). Thus, glutamate taken up into astrocytes through glutamate transporters (GLT) after synaptic release stimulates glycolysis and lactate production in astrocytes. In this process, glutamate uptake into astrocytes and the resulting increase in intracellular Na^+^ have been identified as a key signal coupling excitatory neural activity to increased glucose utilization (Magistretti et al., 1999; Chatton et al., 2000). Moreover, glutamate stimulates aerobic glycolysis in astrocytes (Pellerin and Magistretti, 1994). This metabolic action of glutamate is mediated by glutamate transporters, and involves the activity of Na^+^/K^+^ ATPase in astrocytes (Pellerin and Magistretti, 1997). In fact, local perfusion of the glutamate reuptake inhibitor dihydrokainate (DHK) has been reported to attenuated FS-induced increment eLAC in the mPFC (Uehara et al., 2007b). On the other hand, activation of NMDA-Rs increases eLAC in the mPFC of rodents (Kuhr and Korf, 1988; Schasfoort et al., 1988). These authors reported that infusion of NMDA (10 mM) or kainic acid (0.5 mM) for 1 min resulted in an transient increase in extracellular lactate concentrations, which lasted several minutes longer than the drug administration period. Furthermore, MK-801, as well as other NMDA-R antagonists, may produce disinhibition of glutamate and γ-aminobutyric acid (GABA) or other inhibitory inputs to the glutamate-containing neurons, thereby enhancing the firing rate of these glutamatergic neurons. Such disinhibition may occur locally at the PFC and/or at regions with ascending glutamatergic projections to the PFC (Moghaddam et al., 1997). These findings suggest that systemic MK-801 administration increases glutamate levels in the synaptic cleft, and prolong eLAC elevation in the mPFC.

We previously reported that the long-term effect of transient MK-801 treatment in neonatal periods on lactate metabolism in the mPFC of young adult rats (Uehara et al., 2012a). According to the study, transient MK-801 treatment in neonatal stage suppressed FS stress-induced eLAC elevation in the mPFC without changes in the basal levels. Moreover, Neonatal NMDA-R blockade-induced attenuation of lactate response was ameliorated by 5-HT_1A_ receptor stimulations for 14 days in young adult stage. These findings were completely *opposite* effect to young adult rats treated repeatedly with MK-801 in this study. It is possible that the opposite effect on eLAC reactions to FS was based on the timing of MK-801 administration. Administration of NMDA-R antagonists in the late fetal or early postnatal periods enhances neuronal apoptosis (Ikonomidou et al., 1999; Beninger et al., 2002; Harris et al., 2003). PCP administration in the neonatal stage has been shown to induce apoptotic changes, gliosis (Wang et al., 2001, 2003) and to reduce spine density (Nakatani-Pawlak et al., 2009) in the frontal cortex. These findings suggest that neonatal blockade of NMDA-R produced morphological changes and then reduction of the production source and/or dysfunction of the astrocyte-neuron lactate shuttle system. Furthermore, neonatal blockade of the NMDA-R led to up-regulation of the NR1 receptor subunit in the frontal cortex (Wang et al., 1999, 2001; Du Bois and Huang, 2007). We reported neonatal MK-801 administration reduced the number of parvalbumin-positive GABA interneurons in the mPFC (Uehara et al., 2012b,c). Based on these findings, it is possible that neonatal MK-801 treatment decreased glutamate release in the mPFC, leading to the reduction of eLAC. On the other hand, low doses of MK-801 (<3 mg/kg) induced transient vacuolization in cortical layers III and IV of adult rodents, which was maximal 12 h after the drug and then gradually disappeared during the next 12–18 h (Olney et al., 1989). Therefore, it is assumed that the dose of MK-801 (0.1 mg/kg) in this study may have produced little morphological changes.

Co-administration of tandospirone, a 5-HT_1A_ receptor partial agonist, attenuated prolonged eLAC elevation in the mPFC induced by MK-801 (Figure 3C). Acute treatment with tandospirone has been shown to attenuate the eLAC elevation by FS (Uehara et al., 2006). 5-HT_1A_ agonists have been shown to inhibit potassium-evoked glutamate release *in vitro* (Mauler et al., 2001) while MK-801 increases glutamate in the mPFC *in vivo* (Lopez-Gil et al., 2009). 5-HT_1A_ receptors are also involved in the modulation of excitatory glutamatergic neurotransmission, since their activation reduces NMDA-mediated currents in PFC neurons (Zhong et al., 2008). Moreover, 5-HT_1A_ agonists block the MK-801-induced increase in 5-HT concentrations in the mPFC (Lopez-Gil et al., 2009). Therefore, it is possible that tandospirone shortens the period of eLAC elevation through inhibition of glutamate release and serotonergic transmissions.

Unexpectedly, 4-day treatment with tandospirone (5.0 mg/kg/day, i.p.) did not suppress FS-induced eLAC elevation in the mPFC of saline-treated rats, in contrast to our previous observation with tandospirone at 2.0 mg/kg (Uehara et al., 2006). Moreover, the drug at 0.1, 1.0, or 5.0 mg/kg attenuated FS-induced eLAC increment (Uehara et al., 2012a), although last injection was 24 h before eLAC measurement.

Chronic administration of 5-HT_1A_ agonists has been shown to desensitize 5-HT_1A_ auto receptors in the raphe nucleus, but not frontal cortex (Sim-Selley et al., 2000; Hensler et al., 2003). After sustained treatment with these agents, 5-HT neurons in the raphe nucleus gradually recover their baseline firing rates and become normalized in 14 days (Blier and De Montigny, 1987; Godbout et al., 1991; Blier and Ward, 2003). Treatment with tandospirone for 2 days (10 mg/kg/day, s.c.), markedly reduces the firing activity of 5-HT neurons of the dorsal raphe, followed by partial recovery after 7 days and complete recovery after 14 days of tandospirone administration (Godbout et al., 1991). Therefore, 4-day treatment with tandospirone may decrease 5-HT release into the synaptic cleft in the mPFC. On the other hand, postsynaptic 5-HT_1A_ receptor stimulation inhibits NMDA antagonist-induced glutamate release in the mPFC (Calcagno et al., 2006) and potassium-evoked lactate release *in vitro* (Mauler et al., 2001). Taken together, the discrepant results in our reports, above, may be explained by the balance between autoreceptor desensitization in the raphe nucleus and intensity of stimulation of post-synaptic receptors in the mPFC.

In summary, stimulation of 5-HT_1A_ receptors normalized changes of lactate metabolism in the mPFC of rats exposed to repeated NMDA-R injections. Our findings provide a possible mechanism underlying the clinical observations that tandospirone improves cognitive deficits in patients with schizophrenia (Sumiyoshi et al., 2000, 2001a,b). Importantly, lactate metabolism may provide a novel probe for the development of therapeutics for cognitive impairment of schizophrenia (Uehara and Sumiyoshi, 2013).

## Author contributions

Takashi Uehara and Tomiki Sumiyoshi designed the study and wrote the protocol. Takashi Uehara and Tomiki Sumiyoshi oversaw data collection. Takashi Uehara and Tadasu Matsuoka made animal models. Takashi Uehara undertook analysis of microdialysis data. Tomiki Sumiyoshi provided consultation regarding all aspects of the study. Takashi Uehara wrote the first draft of the manuscript. All authors contributed to and have approved the final manuscript.

### Conflict of interest statement

The authors declare that the research was conducted in the absence of any commercial or financial relationships that could be construed as a potential conflict of interest.
